# Biosensor-based spatial and developmental mapping of maize leaf glutamine at vein-level resolution in response to different nitrogen rates and uptake/assimilation durations

**DOI:** 10.1186/s12870-016-0918-x

**Published:** 2016-10-21

**Authors:** Travis L. Goron, Manish N. Raizada

**Affiliations:** Department of Plant Agriculture, University of Guelph, 50 Stone Road East, Guelph, ON N1G 2W1 Canada

**Keywords:** Maize, Biosensor, Nitrogen, Glutamine, Leaf, Metabolomics, Longitudinal vein, Transverse vein, Nitrogen use efficiency, Imaging

## Abstract

**Background:**

The amino acid glutamine (Gln) is a primary transport form of nitrogen in vasculature following root uptake, critical for the location/timing of growth in maize and other cereals. Analytical chemistry methods do not permit *in situ* analysis of Gln, including visualization within the vascular network. Their cost and tissue requirement are barriers to exploring the complexity of Gln dynamics. We previously reported a biosensor, *GlnLux*, which can measure relative Gln levels inexpensively with tiny amounts of tissue.

**Results:**

Here, maize seedlings were given different N rates for multiple uptake/assimilation durations, after which > 1500 leaf disk extracts were analyzed. A second technique permitted *in situ* imaging of Gln for all leaves sampled simultaneously. We demonstrate that multifactorial interactions govern Gln accumulation involving position within each leaf (mediolateral/proximodistal), location of leaves along the shoot axis, N rate, and uptake duration. *In situ* imaging localized Gln in leaf veins for the first time. A novel hypothesis is that leaf Gln may flow along preferential vascular routes, for example in response to mechanical damage or metabolic needs.

**Conclusions:**

The *GlnLux* technology enabled the most detailed map of relative Gln accumulation in any plant, and the first report of *in situ* Gln at vein-level resolution. The technology might be used with any plant species in a similar manner.

**Electronic supplementary material:**

The online version of this article (doi:10.1186/s12870-016-0918-x) contains supplementary material, which is available to authorized users.

## Background

Nitrogen (N) contributes approximately 2 % of dry plant matter and is the most important nutrient for plants by quantity [1, 2]. N is crucial for the biosynthesis of amino acids, proteins, nucleic acids, chlorophyll and secondary metabolites, all of which are essential macromolecules [3]. Plant roots absorb N primarily as ammonium (NH_4_^+^) or nitrate (NO_3_^−^). The NO_3_^−^ portion is reduced to NH_4_^+^ by a combination of nitrate reductase and nitrite reductase (NR, NiR). Free NH_4_^+^ is then assimilated into a pool of amino acids by the glutamine synthetase (GS)/GOGAT cycle, and used for a wide variety of biological processes including protein synthesis in young, expanding tissue [1]. In maize (*Zea mays* L.), nitrogen assimilation occurs in both roots and shoots similar to other species [4–7], and depending on the environmental conditions [3, 8]. One of the primary assimilatory amino acids, glutamine (Gln), displays immediate and rapid increase in leaves following N application to roots as nitrate and/or ammonium, and drastic differences in concentration depending on the developmental stage [9, 10]. As such, the concentration and localization of Gln may serve as a convenient proxy to study developmental-dependent dynamics of N assimilation [11, 12].

Although many studies of N uptake and assimilation have been conducted on a whole-field scale [13–16] or plant scale [17–20], investigations of N spatial, developmental and temporal dynamics within individual tissues are limited. In particular, high-resolution metabolic maps of N dynamics in young maize shoot tissue are extremely scarce. The maize shoot encompasses an entire developmental gradient of sequentially initiating leaves [21, 22], and at any time-point a single plant possesses leaves of different ages corresponding to order of emergence, with the lowest leaf being the oldest [21]. Additionally, leaf growth occurs in two dimensions, along the proximodistal and mediolateral axes. In maize, growth along the proximodistal leaf axis occurs basipetally: young sink tissue initiates near the base of the leaf blade (ligule), and differentiates towards the leaf tip [23, 24]. The mediolateral axis in maize is bilaterally symmetrical around the midvein. Additional longitudinal veins run parallel to the midvein and are interconnected by narrower transverse veins [25]. Following uptake by roots, N and assimilates are transported over time through these developmental and spatial gradients, in part employing the vein network.

In recent years, several authors have beautifully characterized metabolic, proteomic, and transcriptomic changes along a one-dimensional basipetal gradient in a single maize leaf [23, 24, 26, 27]. These studies utilized analytical chemistry techniques to examine N assimilates [23, 24, 26, 27]. A limitation of these analytic methods is that they do not permit *in situ* spatial analyses of metabolites and hence offer limited two-dimensional spatial resolution and overlook the critical vein network. Furthermore, when N is taken up by roots, N assimilates accumulate based not only on two-dimensional spatial gradients within a tissue, but also on tissue position and age (growing versus mature), relationships to other source/sink tissues, available N concentration, and time for uptake, assimilation and migration [3]. Elucidating these multifactorial interactions would necessitate diagnostic technologies that are simple, low-cost and require minimal tissue in order to permit measurements of Gln and other N assimilates with thousands of data points.

Whole-cell biosensors are engineered microbes that detect analytes, amplify the signal and emit a measurable output such as fluorescence or luminescence [28]. Previously, we reported a biosensor for Gln, named *GlnLux,* based on an *Escherichia coli* Gln auxotroph which luminesces when exogenous, free Gln is supplied [12]. We demonstrated that when *GlnLux* cells are exposed to Gln from maize tissue extracts, they multiply and release photons due to the presence of a constitutively expressing *lux* operon. The photons can be measured using a luminometer. We demonstrated that *GlnLux* output from maize leaf disk extracts highly correlates to high performance liquid chromatography (HPLC) measurements of Gln [12]. The technology was shown to be sensitive to < 1 nM Gln, suggesting it could be used for accurate, high-throughput Gln mapping using 96-well plates. To image Gln *in situ* directly from entire organs, they may be freeze-thawed to cause Gln leakage due to cellular damage, and placed on agar pre-embedded with *GlnLux* cells (*GlnLux* agar). This strategy ensures equal access of the tissue surface to biosensor cells, as opposed to direct incorporation which is impractical. Photons are released from the biosensor cells in proximity to the plant cells which can then be imaged using a photon capture charge coupled device (CCD) camera [12].

The primary objective of this study was to use the *GlnLux* biosensor technologies to conduct detailed spatial and developmental gradient mapping of maize leaf Gln in response to different N rates and uptake/assimilation durations. The second objective was to determine if *GlnLux* *in situ* imaging could achieve resolution to the leaf vein level.

## Methods

### Plant growth conditions

*Zea mays* L. hybrid CG60 X CG102 [29] seed was used for all experiments. Seeds were surface-sterilized by soaking 4 min in 70 % ethanol solution, 2 min in 4 % NaClO, followed by washing five times in sterile double distilled (dd) H_2_O. Seeds were germinated in 18-cell (two per cell, 8.5×8.5×9 cm) growth trays of Turface® (Profile Products, Buffalo Grove, USA), a baked-clay gravel with extremely low background levels of nitrogen (N). In previous experiments [30, 31] the gravel was found to contain 0.053 % N, of which only a fraction is available for plant uptake; N-free nutrient solution soaked with the Turface® gravel for 24 h was found to contain only 1.42 mg/L total N, equivalent to 0.1 mM. Growth flats were placed into plastic sub-irrigation trays (51×25.5×6 cm) containing 2 L ddH_2_O with no additional nutrients. For germination, the trays were initially placed in darkness in a laboratory cabinet at room temperature until plant emergence, thinned to one plant per cell, and arranged (completely randomized design, CRD) in a greenhouse with the following growing conditions: 28 °C/20 °C day/night (16 h/8 h), with 1000 W high pressure sodium and 1000 W metal halide lamps supplemented with GroLux bulbs, resulting in an average light intensity range of 803–1026 μmol m^−2^ s^−1^ (canopy level at noon). Plants were randomized daily and watered with ddH_2_O as needed.

### Relative measurements of glutamine from leaf disk extracts

Twelve days after sowing (DAS), sub-irrigation trays were emptied of remaining ddH_2_O. Plants were supplied with one of six different modified Hoagland’s nutrient solutions consisting of 0.1 mM K_2_SO_4_, 1.0 mM KCl, 2 mM KH_2_PO_4_, 1 mM MgSO_4_·7H_2_O, 0.03 g/L chelated micronutrients (10046, Plant Products, Leamington, Canada) and either 0, 2, 5, 10, 15, or 20 mM total N provided as NH_4_NO_3_. Each nutrient solution (1.5 L) was poured into the sub-irrigation trays, with an additional 30 ml applied near the base of each plant.

At various time-points after nutrient application (1, 6, 18, 12, and 24 h; starting at 9:30 AM, 2:30 PM, 8:30 PM, 2:30 AM, and 8:30 AM respectively), sampling was performed on leaves 1, 2, and 3, as defined by their order of emergence. Leaf tissue disks (6.35 mm in diameter) were harvested with a hand punch tool (235270975, Fiskar’s Brands Inc., Middleton, USA) at equally spaced intervals along the mid-vein, extending from the ligule region to the leaf tip in leaves 1 and 2. Because leaf 3 was still expanding, harvest of leaf disks extended from where the leaf exited the whorl to the tip. All tissue was frozen immediately in liquid N_2_, and stored at −80 °C. Three, five, and four different positions were harvested from leaves 1, 2 and 3 respectively. Four plants (replicates) were sampled for each time/nitrogen combination, and the most informative treatments were repeated in an independent trial.

**Fig. 1:**
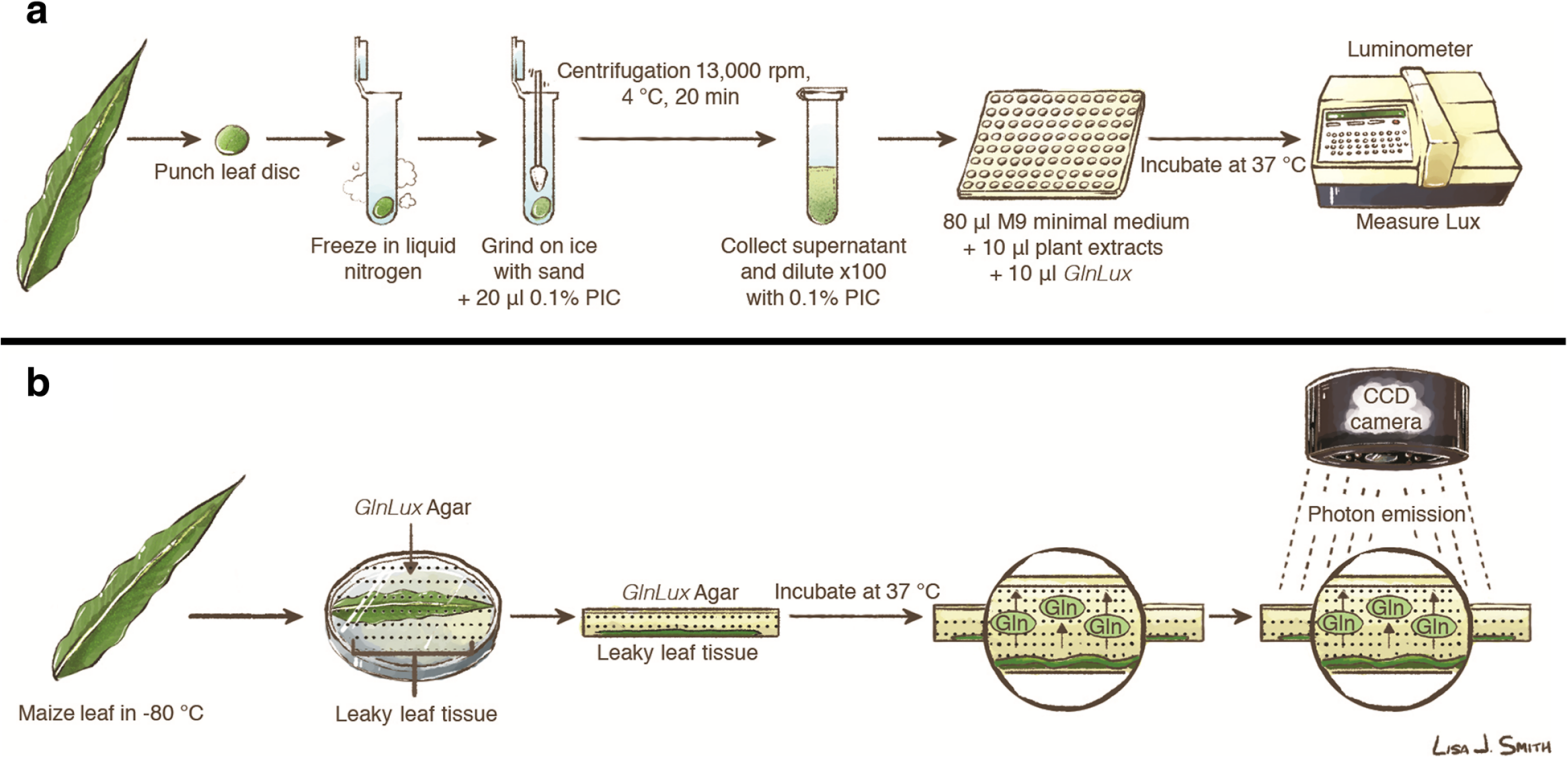
Schematic images of the *GlnLux* protocols. Overview of the *Glnlux* liquid assay using extracts of leaf punches incubated with *GlnLux* biosensor cells in 96-well plates and measured using a luminometer (**a**). Overview of the *GlnLux* *in situ* imaging assay (**b**). Leaves are frozen at −80 °C and thawed at room temperature for 30 s to cause Gln leakage. Leaves are pressed down on agar pre-embedded with *GlnLux* cells, referred to as *GlnLux* agar. Plates are inverted and incubated for 2.5 h and then imaged for 1000 s using a luminescence imaging system. PIC, protease inhibition cocktail. Images are courtesy of Lisa Smith (University of Guelph), and can be re-used under the Creative Commons BY license

Leaf tissue disks were analyzed for glutamine (Gln) content with the *GlnLux* biosensor as previously described [12] with some modifications (Fig. 1a). Leaf disks were homogenized with a pellet pestle in a mixture of sterile sand and 20 μl 0.1 % chilled protease inhibition cocktail (PIC) (P9599-1ml, Sigma-Aldrich, St. Louis, USA), and centrifuged (model 5415R, Eppendorf, Hauppauge, USA; 4 °C, 20 min, 13 200 rpm). The resulting plant tissue extract supernatant was diluted 100-fold in 0.1 % PIC and stored overnight at −20 °C until analysis.

Concurrently, *GlnLux* biosensor cells were cultured for 16 h in Luria Broth (LB) (37 °C) with shaking (245 rpm). Biosensor cells were then pelleted (2500 rpm, 10 min) and washed with M9 minimal growth media (DF0485170, BD, USA) three times. Cells were re-suspended in M9 media (OD_595_ = 0.025) and incubated for 16 h (37 °C, 245 rpm) to deplete endogenous Gln. All media were supplemented with 50 μg/ml kanamycin and 100 μg/ml carbenicillin, as *GlnLux* contains Kan^r^ and Amp^r^ resistance genes [12].

Each leaf disk extract (10 μl) was combined with 10 μl prepared *GlnLux* cells and 80 μl M9 in white, flat bottom 96-well plates (07-200-589, Corning Inc., Corning, USA). A negative control of 10 μl 0.1 % PIC in place of extract was also included on each 96-well plate for subtractive normalization of the luminescence data. Plates were incubated for 2 h to allow biosensor activation, then luminescence output was quantified using a 96-well luminometer (MicroLumatPlus, Berthold Technologies, Bad Wildbad, Germany) (37 °C, 1 s photon capture per well).

Normalized luminometer data (raw outputs – negative control) was plotted against the duration of N uptake/assimilation, and against the N application rate. Outliers were identified and removed with ROUT, Q = 1 % [32]. Means were compared with the Holm-Šídák method [33–35], or Dunnett’s multiple means comparison [36] at *P* < 0.05 as indicated in the figure legends. Kruskal-Wallis tests with Dunn’s multiple means comparisons were used where data displayed non-normality, as identified with Bartlett’s test [37–39]. All statistical analyses were performed in GraphPad Prism 6 (GraphPad Software Inc., San Diego, USA).

### Generating whole-leaf *in situ* images of free glutamine

As above, at 12 DAS, sub-irrigation trays were emptied of remaining ddH_2_O. Plants were then supplied with 0 or 20 mM total N (NH_4_NO_3_) provided as modified Hoagland’s nutrient solution (as described above). Again, 1.5 L of nutrient solution was poured into each sub-irrigation tray, and 30 ml near the base of each plant.

Leaves were harvested after 1 h (starting at 9:30 AM), 12 h (8:30 PM) and 24 h (8:30 AM) of N uptake/assimilation. Harvesting of leaf 1 consisted of removing the entire leaf at the ligule. For the younger leaves, as the ligules had not yet developed, leaves 2 and 3 were cut from the plant where the leaf blade curled in upon itself to meet the stem. Three replicates were harvested per treatment combination, frozen immediately in liquid N_2_, and stored at −80 °C until imaging.

Images of free Gln within maize leaf tissue were generated with *GlnLux* solid agar media as previously described [12] with modifications (Fig. 1b). Briefly, *GlnLux* biosensor cells were cultured for 16 h (37 °C, 245 rpm) in LB broth supplemented with 0.2 mM Gln, 4.0 mM glucose, 50 μg/ml kanamycin and 100 μg/ml carbenicillin. Cells were then centrifuged (2500 rpm, 10 min), re-suspended in 0.01 M potassium phosphate buffer (pH 7.0) and washed two more times. Cells were suspended in M9 medium (OD_595_ of 1.0). *GlnLux* solid agar media was prepared by combining the *GlnLux* culture (10 % v/v) with concentrated M9 medium containing 10 g/L Bacto agar pre-cooled (to 42 °C), and pouring this mixture into sterile 150×15 mm Petri dishes. *GlnLux* solid agar media plates were stored at 4 °C overnight prior to use. Frozen leaves were thawed at room temperature for 30 s and pressed into the *GlnLux* agar (pre-incubated at room temperature). Plates were inverted, incubated (37 °C, 2.5 h), and imaged with a charge-coupled-device (CCD) chip camera (7383–0007, Princeton Instruments, Trenton, USA) pre-cooled to −100 °C for a 1000 s exposure. Incubation and imaging of plates were staggered to ensure that conditions across replicates were constant. However, to negate the potential effects of slight incubation length differences (on the scale of seconds) *in situ* image standardization was performed across plates in WinView (version 2.5.16.5, Princeton Instruments, Trenton, USA) by adjusting image intensity according to the signal produced by a disk of agar (2.4 % agar in water, radius = 3 mm) containing 1 × 10^−2^ M Gln pressed into each plate at the time of leaf placement. This effect was examined for its potential to confound results by comparison of the standard disk image intensity to that of leaves pooled across N treatments, with *F* tests at *P* < 0.05 (GraphPad Prism 6, GraphPad Software Inc.)

### Investigating the effect of Gln diffusion on whole-leaf *in situ* images

To examine Gln diffusion through *GlnLux* agar, luminescence output from leaves was visualized over multiple, consecutive incubation intervals. Plants were initially germinated and grown with only ddH_2_O in Turface® gravel until they were at the same growth stage as the main experiments. Hoagland’s solution containing 20 mM N was then provided for 2 h, after which plants were moved back to N-free solution for a further 10 h. Leaves 1, 2, and 3 were harvested and placed on *GlnLux* agar alongside disk standards of Gln (0, 3.125 × 10^−4^, 6.250 × 10^−4^, 1.250 × 10^−3^, 2.500 × 10^−3^, 5.000 × 10^−3^, 1 × 10^−2^ M, left to right; volume = 51 μl, radius = 3 mm). Plates were imaged once before incubation, and then incubated at 37 °C for intervals of 1000 s with imaging following each interval. Plates were incubated a further 6.5 h and imaged. All images were captured with a 1000 s exposure and standardized to a range of 1000–6000 light intensity units in WinView (version 2.5.16.5, Princeton Instruments, Trenton, USA).

To determine the effect that Gln diffusion through the *GlnLux* agar imposes on vein-level resolution, the diameters of midveins, longitudinal, and transverse leaf vein tissues were quantified with NIS-Elements (version 4.51, Nikon Instruments, Tokyo, Japan) following 4x brightfield microscopy (Nikon Eclipse 50i, Nikon Instruments). Diameters of longitudinal and transverse leaf vein tissues from whole-leaf *in situ* images were quantified with ImageJ (version 1.50i, NIH, Bethesda, USA) for comparison against microscopy with the Holm-Šídák test at *P* < 0.05 (GraphPad Prism 6, GraphPad Software Inc.). The veins of three biological replicates were quantified using both microscopy and *in situ* images.

It was postulated that differing tissue thicknesses may impact the luminescence output of *in situ* images. Two experiments were conducted to investigate this possibility:

i) Three sets of agar Gln disks with different heights/volumes were prepared, scaling linearly (*h* = 1.8, 3.6, 5.4 mm; *V* = 51, 102, 153 μl. Radius was held constant at 3 mm). The molarity of Gln within the standards was held constant across the three different height/volume levels (0, 3.125 × 10^−4^, 6.250 × 10^−4^, 1.250 × 10^−3^, 2.500 × 10^−3^, 5.000 × 10^−3^, 1 × 10^−2^ M Gln). Image standardization was applied using WinView software (version 2.5.16.5, Princeton Instruments) (1000 s exposure, 1000–6000 light intensity units) after 2.5 and 6 h.
ii) Three sets of agar Gln disks with different heights/volumes were prepared, scaling linearly as above. However, total moles of Gln within the standards was held constant across the three different height/volume levels (0, 15.94, 31.87, 63.75, 127.5, 255.0, 510.0 nmol). Image standardization was applied using WinView software (version 2.5.16.5, Princeton Instruments) (1000 s exposure, 1000–6000 light intensity units) after 2.5 and 6 h.

## Results

### Gradients of leaf glutamine occur in response to the rate and duration of nitrogen uptake/assimilation

**Fig. 2:**
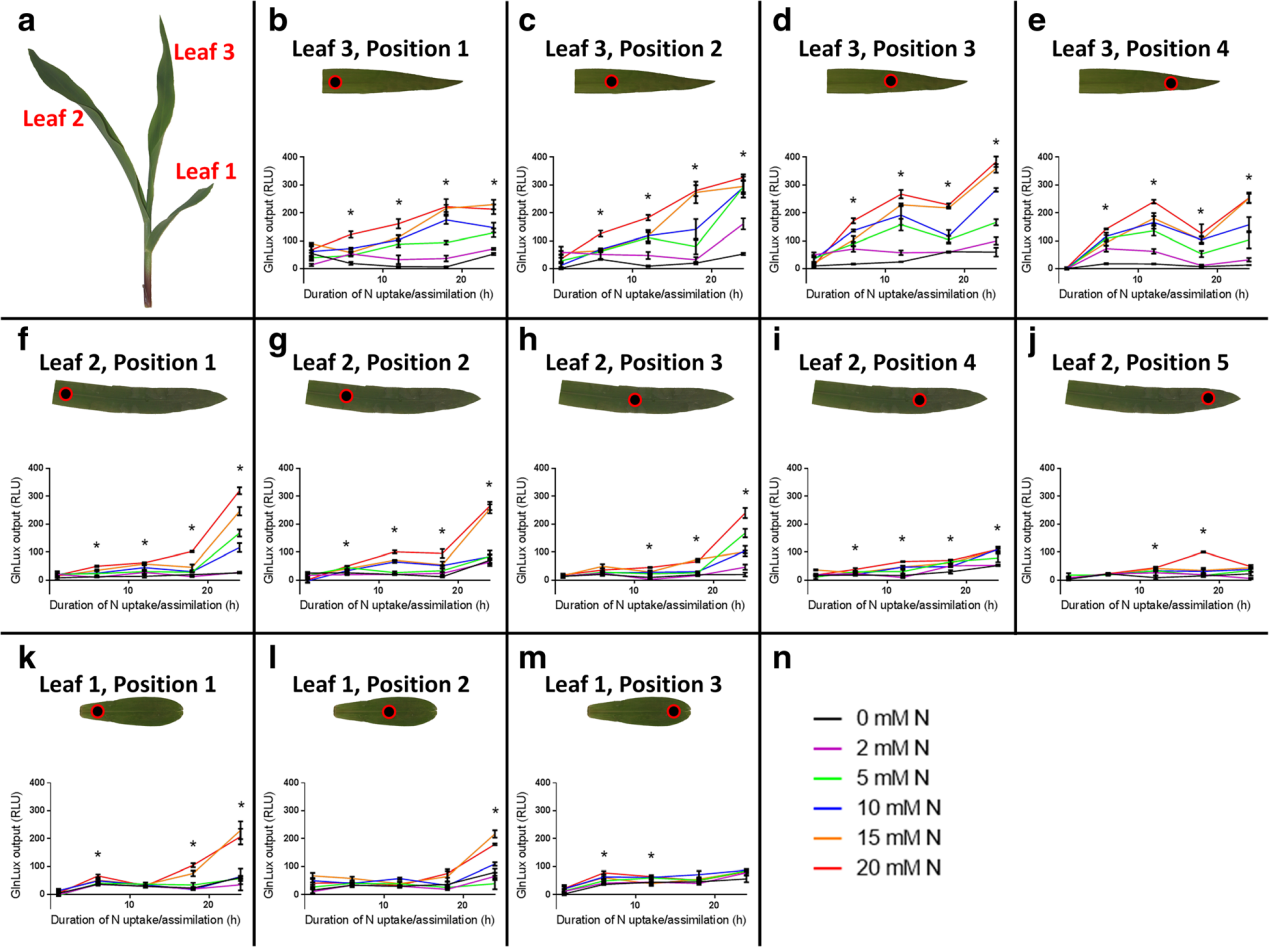
Gradients of *GlnLux* output of leaves of maize seedlings using the *GlnLux* leaf disk assay. Leaves 1, 2 and 3 were sampled (**a**). Leaf 3 was assayed at positions 1 (**b**), 2 (**c**), 3 (**d**), and 4 (**e**), extending from the leaf base to leaf tip. Leaf 2 was assayed at positions 1 (**f**), 2 (**g**), 3 (**h**), 4 (**i**), and 5 (**j**). Leaf 1 was assayed at positions 1 (**k**), 2 (**l**), and 3 (**m**). Plants had not been provided with N from germination for a period of 12 days, at which time modified Hoagland’s solution containing 0, 2, 5, 10, 15 or 20 mM N (**n**) was applied. Plants were allowed different durations (1, 6, 12, 18 or 24 h) of N uptake/assimilation, after which tissue disks were harvested. Means of 3–4 replicates +/− SEM are shown. RLU, relative light units intercepted by the luminometer in a one second interval per well. Asterisks indicate significant differences between the 0 mM (black lines) and 20 mM (red lines) N treatments, based on the Holm-Šídák test at *P* < 0.05. The data is displayed to highlight the N-uptake/assimilation gradient. The N rate response gradient is highlighted in Additional file 1: Figure S1. The two datasets are the same. Shown is Trial 1. For Trial 2, see Additional file 2: Figure S2 and Additional file 3: Figure S3

**Fig. 3:**
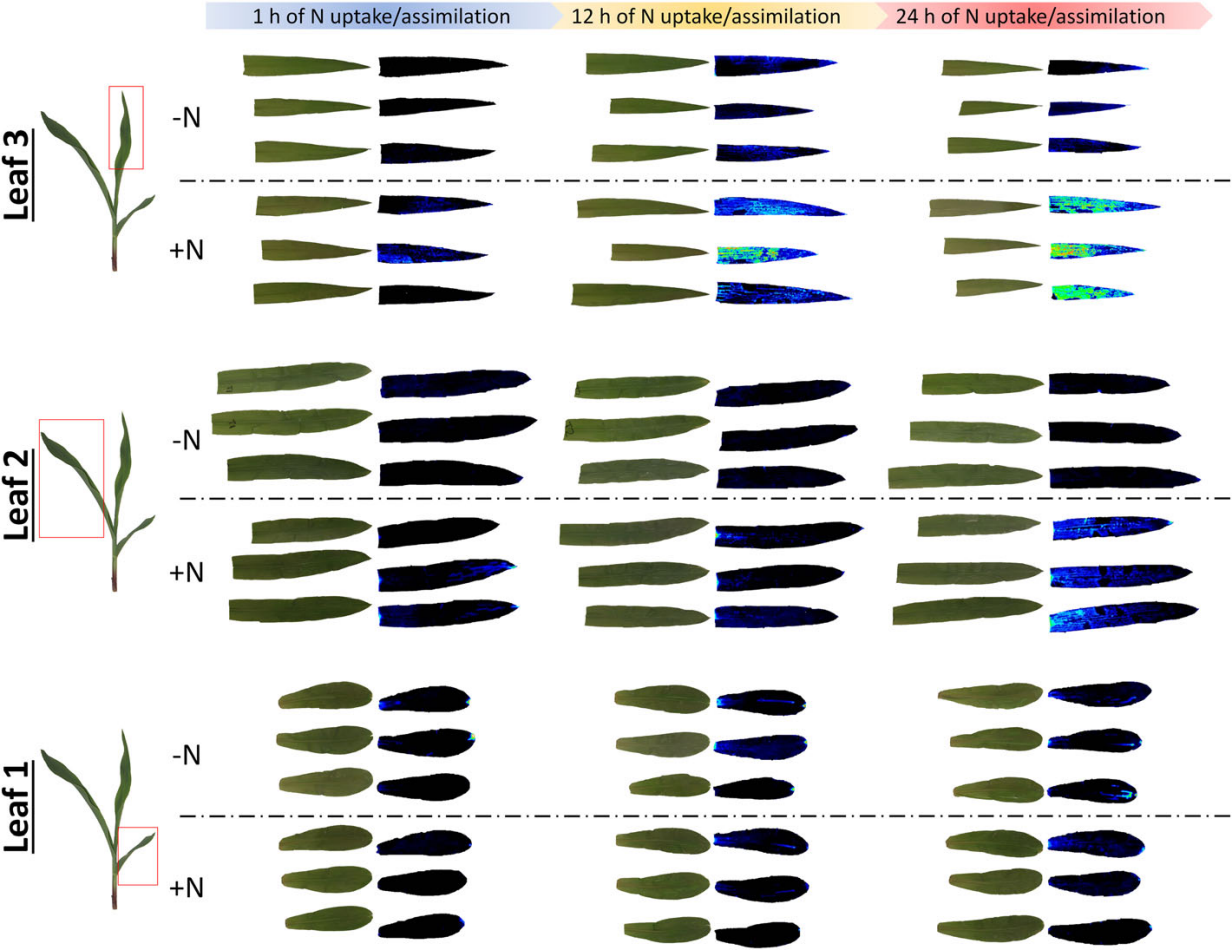
Gradients of *GlnLux* output of seedling leaves of maize seedlings using *in situ* imaging. Plants were initially treated with only water, and then at day 12, they were exposed to Hoagland’s nutrients solution containing either 0 mM N (−N) or 20 mM N (+N) for 1, 12, or 24 h, after which the leaves were harvested and placed on *GlnLux* agar. *GlnLux* images are shown directly beside light images of each leaf. Red-yellow-green indicates diminishing *GlnLux* response, and black indicates absence of *GlnLux* output. Three replicates of each treatment combination are displayed vertically

After a 12-day N starvation period, plants were provided with varying N concentrations ranging from 0–20 mM for N uptake/assimilation periods spanning 1 - 24 h before sample collection (Fig. 2). Leaves were then analyzed for relative free glutamine (Gln) levels using the leaf punch *GlnLux* assay (Figs. 1a and 2 and Additional file 1: Figure S1). Generally, for a given spatial position, increased N rate and duration of N uptake/assimilation induced greater *GlnLux* output (Fig. 2 and Additional file 1: Figure S1), but interestingly this varied by leaf position (see below). A smaller independent second trial confirmed these trends (Additional file 2: Figure S2). Additionally, *in situ* images of Gln accumulation in whole leaves were generated by placing them on *GlnLux* agar (Figs. 1b and 3). When plants were provided with either 0 (−N) or 20 (+N) mM total N, leaves showed similar trends as were observed using the leaf punch assay (Fig. 3). This was especially evident in leaves 2 and 3 (Fig. 3).

### Glutamine levels display developmental gradients along the shoot axis and leaf proximodistal axis, and symmetry along the mediolateral axis

Using the leaf punch assay, *GlnLux* output showed dependency on leaf age (order of emergence) along the shoot axis (Fig. 2 and Additional file 1: Figure S1). At equivalent relative sampling positions, the oldest leaf (leaf 1) generally displayed the lowest output levels, while leaves 2 and 3 displayed progressively higher output based on the *GlnLux* leaf punch assay (Fig 2 and Additional file 1: Figure S1). Trial 2 was consistent with these results (Additional file 3: Figure S3). The trend was also clearly observed in the *in situ* images which showed dramatically increased luminescence output in leaf 3 compared to leaf 1 for + N treated plants, with leaf 2 showing an intermediate response (Fig. 3).

The leaf punch assay showed that *GlnLux* output was dependent upon the sampling position along the proximodistal axis within a leaf (Fig. 2 and Additional file 1: Figure S1). Specifically, positions nearing the base of a leaf showed increasing responses to N rate and duration compared to the tip (a basipetal gradient) which was especially clear in leaves 1 and 2, but less pronounced in leaf 3 (Fig. 2 and Additional file 1: Figure S1). Trial 2 was consistent with these results (Additional file 3: Figure S3). The *in situ* images of Gln accumulation similarly showed greatest *GlnLux* output towards the base of leaves 2 and 3 in + N treated plants. Leaf 1 of N treated plants showed low and variable *GlnLux* output.

**Fig. 4:**
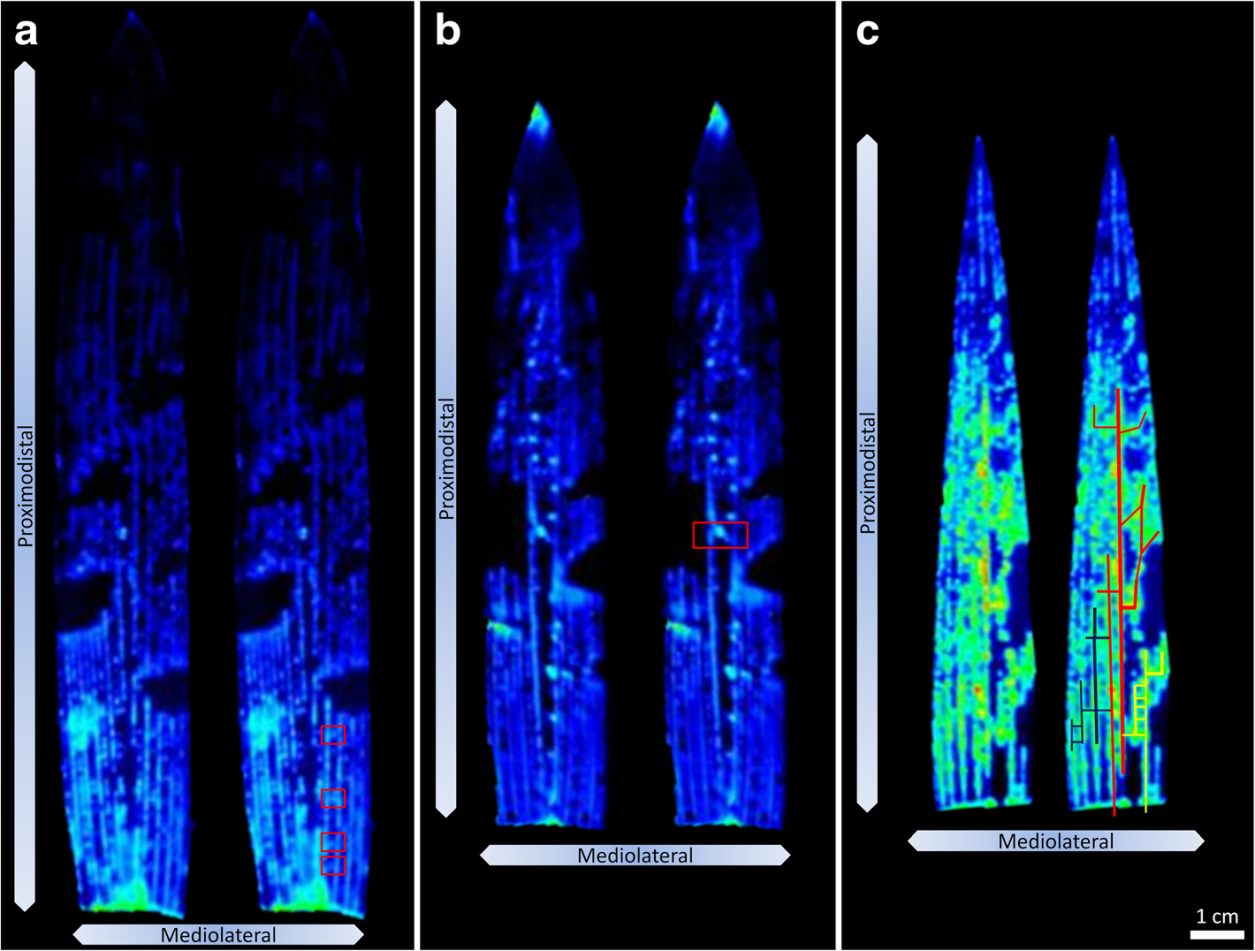
*In situ* images of maize leaves following N fertilization reveal vein-level resolution of *GlnLux* output. Shown are magnified images from Fig. 3. The images highlight transverse veins in leaf 2 (**a**, **b**, *red boxes*). Potential vascular networks of Gln movement through longitudinal and transverse veins are shown in leaf 3 (**c**, *coloured tracings*). In each panel, duplicate images of a single leaf are shown, with highlights of vein-level details to the right. Arrows indicate directions of the proximodistal and mediolateral axes

In general, there was symmetry in *GlnLux* output along the mediolateral axis from the midvein to the leaf edges (Figs. 3 and 4). However, asymmetric patches of high and low intensity were observed (Fig. 4).

### *In situGlnLux* leaf images localize Gln to vein-level resolution

The *in situ* images attained vein-level resolution, revealing fine-scale details of Gln localization (Fig. 4). Luminescence could be observed in both the large and small parallel longitudinal veins along the proximodistal axis (Fig. 4a, b). In the transverse veins (along the mediolateral axis) that connect the longitudinal veins, luminescence could also be observed (Fig. 4a). Intense luminescence was sometimes observed along apparently connected networks of longitudinal veins and transverse veins (coloured tracing, Fig. 4c). Although there was clear separation between leaf veins, it was determined that the level of resolution attained with *GlnLux* imaging is less than standard light microscopy (Additional file 4: Figure S4) likely due to a combination of photon scatter and limited Gln diffusion in the agar (Additional file 5: Figure S5).

## Discussion

### High-resolution *GlnLux* methodologies permit measurements and visualization of single-factor gradients

In this study, the high sensitivity of the *GlnLux* biosensor (< 1 nM) [12] permitted small leaf disks to be used for relative Gln measurements which facilitated detailed spatial leaf analysis. The low cost (~$1 USD per sample) and high-throughput nature of the protocol enabled > 1500 samples to be processed to provide a detailed spatial/temporal map of relative Gln in a young maize shoot. Leaf disks sampled along the midvein displayed increasing gradients of *GlnLux* output based on increased N application rate and duration of N uptake/assimilation, and position along the leaf proximodistal axis and shoot axis (Fig. 2 and Additional file 1: Figure S1). The more intensive technique, *Glnlux* imaging, permitted these gradients to be visualized *in situ* in two-dimensions along both the leaf proximodistal and mediolateral axes (Figs. 3 and 4) and for the first time at vein-level resolution (Fig. 4).

Previous studies have observed a rapid appearance of Gln in maize leaf tissue following N application [10, 40]. In leaf 2 harvested from young maize plants, Gln was shown to accumulate in a basipetal gradient peaking at the base [9]. More recently, highly-detailed analyses revealed that Gln and transcripts related to protein metabolism displayed a similar gradient in leaf 3 [24, 26, 27].

The progressively higher *GlnLux* output along the shoot axis (Fig. 3) likely indicates preferential shuttling of assimilatory metabolites into young, photosynthetic, growing tissue [6, 41, 42]. Alternatively, there may be fundamental differences in anatomy (with implications on underlying physiology) between leaves 1–3, though they are classified within the same, embryonic phase of development [43–45].

### *GlnLux* methodologies permit analysis of complex interactions

The studies detailed above concerning the basipetal gradient [9, 24, 26, 27] were performed on a single maize leaf (leaves 2 or 3). The present report is, to the best of our knowledge, the first time such analysis has been performed on different leaves sampled simultaneously on the same plant following application of multiple N rates and durations of uptake/assimilation. When the high-throughput nature of the *GlnLux* leaf punch assay was combined with the detailed *in situ* images, several complex interaction effects could be identified. Specifically, at least five types of interactions were observed: (1) an N rate x N duration interaction (additive, Figs. 2 and 3); (2) an N rate/duration X shoot axis interaction (N preferentially observed in younger leaves, Figs. 2a-j and 3); (3) a shoot axis X leaf proximodistal interaction (leaves 1 and 2 showed greater leaf-basipetal gradients than leaf 3, Figs. 2 and 3); (4) an N rate/duration X leaf proximodistal interaction (only the highest N rates showed a leaf basipetal gradient in leaf 1 in contrast to leaf 2, Figs. 2f, k and 3); (5) and interactions with the leaf mediolateral axis (Figs. 3 and 4) (generally proximodistal and mediolateral axes expression were coincident). These interactions, uncovered using the *GlnLux* technologies, reveal the complexity of N assimilatory dynamics even at the seedling stage.

### *In situ* imaging may uncover preferential routes of Gln movement through the leaf vein network

Current methods utilizing tracer dyes in conjunction with microscopy, x-ray imaging, or magnetic resonance imaging (MRI) are able to observe veins with a fine level of detail [46–50]. However, such analysis is generally restricted to noting the presence/absence of fluid without visualization of specific metabolites. Additionally, most studies are performed on cross sections of stem tissue without providing images of entire leaves. Radioisotope labelling (^13^C or ^15^N) might be used to track movement of metabolites [51, 52], but labelled nitrogen applied to plant roots would be incorporated into other assimilatory metabolites besides free Gln (e.g. other amino acids, protein, chlorophyll).

Here *GlnLux* *in situ* imaging permitted visualization of free Gln at vein-level resolution. Intense *GlnLux* signal was observed in some leaf locations as branch patterns of apparently interconnected longitudinal and transverse leaf veins (coloured tracing, Fig. 4c). Combined, these branches formed a visible network, interspersed with patches of low intensity. The simplest interpretation is that Gln does not diffuse randomly through the vein network but rather can have preferential vascular routes, either to supply local needs or perhaps to bypass spots of vascular damage. The leaves may have been damaged during the procedure, forcing Gln along detours to reach its destination. Specifically, there may have been physical damage to the veins during tissue handling, or cavitation-induced embolisms (air bubbles) might have formed associated with leaf dissection or freezing. All have implications for how plants respond to similar events in the real world [46–48, 53], for example, associated with pest damage, vein callose formation and the formation of ice crystals. *GlnLux* *in situ* imaging should allow future investigation of such hypotheses and may enable a new field of N assimilate research.

Caution must be exercised when analyzing leaf veins with *GlnLux*, as visualized leaf vein tissue (luminescence) has a larger diameter than that quantified with light microscopy, suggesting a degree of diffusion and/or light scatter (Additional file 4: Figure S4). Further examination of luminescence produced by leaves on *GlnLux* agar over multiple consecutive incubation intervals (1000 s) showed insignificant rates of diffusion from leaves as compared to the diffusion-prone Gln agar standards (Additional file 5: Figure S5).

### Limitations of the *GlnLux* technologies

The literature suggests that the concentration range of Gln in maize leaves ranges from 0.06 to 1.1 μmol/g fresh weight [10]. A disadvantage of the *GlnLux* techniques is that only relative and not absolute concentrations of Gln are reported. Inclusion of a standard curve of pure Gln, although highly replicable (Additional file 6: Figure S6 and Additional file 7: Figure S7), is difficult to interpret, in part due to differences in diffusion rates compared to leaf tissue (see above). Furthermore, maize leaves contain many metabolites, at least some of which are likely to impact the growth of the *GlnLux E. coli* cell, perhaps negatively (Additional file 8: Figure S8). However, this negative effect is presumably imposed equally across all tissues and N treatments (visible in Additional file 5: Figure S5).

With respect to the leaf disk assay, the thickness of the leaf vein does vary, potentially adding to the experimental error. Different thicknesses may impose a confounding effect with respect to *GlnLux* *in situ* imaging, similar to that observed with Gln standard disks of different heights/volumes (Additional file 6: Figure S6 and Additional file 7: Figure S7). Tissues of different thickness may have differential rates of diffusion into *GlnLux* media. Image analysis is relative, and hence it is critical to have the treatment and control on the same plate. However, biosensors conceptually similar to *GlnLux* which rely on diffusion of metabolites into agar media have been utilized previously with good correlation of image intensity and independent metabolite quantification [54].

An additional limitation is imposed by the slight curvature of leaf blades, causing incomplete adherence to the *GlnLux* agar, resulting in dark zones (Fig. 3). Furthermore, tissue cracking can result in localized fluid leakage, resulting in artefacts (e.g. Fig. 3, compare light image to *GlnLux* image for top replicate of leaf 1, −N, 12 h). Finally, the finite amount of tissue that can be processed simultaneously in the imaging protocol might be considered a limitation. However, if *GlnLux* plates are properly staggered, plate-to-plate variability does not confound the results (Additional file 9: Table S1).

## Conclusions and future applications

Gln is central to primary N metabolism and therefore potential applications of the *GlnLux* technologies are wide-ranging. The *GlnLux* assays may facilitate detailed metabolic studies, in which high replicate numbers have been suggested as ideal [55–57]. Specifically the assay may be used to probe more complex N dynamics, diurnal rhythms, time-courses of N uptake/assimilation, and to create high-resolution maps of Gln movement. These methods may be applied to other species, as well as to different organs including roots [12]. Additionally, mature plants at later growth stages may be examined. As mature leaves enter senescence it might be of interest to track the remobilization of Gln from shoot tissue to grain, which has been shown to improve nitrogen use efficiency (NUE), defined as the N fertilization requirement per unit of production [58, 59]. The high processing power of the *GlnLux* leaf disk assay may enable screening of genotypes, and breeding for improved NUE by providing links between genetic and phenotypic traits on a fine scale [57, 60].

## Additional files

Additional file 1: Figure S1. Gradients of *GlnLux* output of leaves of maize seedlings using the *GlnLux* leaf disk assay. Leaves 1, 2 and 3 were sampled (a). Leaf 3 was assayed at positions 1 (b), 2 (c), 3 (d), and 4 (e), extending from the leaf base to leaf tip. Leaf 2 was assayed at positions 1 (f), 2 (g), 3 (h), 4 (i), and 5 (j). Leaf 1 was assayed at positions 1 (k), 2 (l), and 3 (m). Plants had not been provided with N from germination for a period of 12 days, at which time modified Hoagland’s solution containing 0, 2, 5, 10, 15 or 20 mM N was applied. Plants were allowed different durations (1, 6, 12, 18 or 24 h) of N uptake/assimilation (n), after which tissue disks were harvested. Means of 3–4 replicates +/− SEM are shown. RLU, relative light units intercepted by the luminometer in a one second interval per well. Asterisks indicate significant differences between the 1 h (black lines) and 24 h (red lines) treatments, at different N application rates, based on the Holm-Šídák test at *P* < 0.05. The data is displayed to highlight the N rate response gradient. The N uptake/assimilation gradient is highlighted in Fig. 2. The two datasets are the same. Shown is Trial 1. For Trial 2, see Additional file 2: Figure S2 and Additional file 3: Figure S3. (PNG 4607 kb)
Additional file 2: Figure S2. Independent trials of *GlnLux* output of maize seedling leaves using the leaf disk assay. Shown is the data from Trial 1 (b, e, h, k) and Trial 2 (c, f, i, l). Two time points of N-uptake and assimilation (1, 24 h) are shown in panels (a-f) to highlight the temporal response gradient, while in panels (g-l) a single time point (24 h) is shown to highlight the N rate response gradient. Leaf 3 was sampled at position 4 (a) in 2013 (b) and 2014 (c), after 1 and 24 h of uptake/assimilation of 0, 2, 5, 10, 15, or 20 mM N. Leaf 1 was sampled at position 1 (d) in 2013 (e) and 2014 (f) after 1 and 24 h of uptake/assimilation. Leaf 3 was sampled at position 1 (g) in 2013 (h) and 2014 (i) after 24 h of uptake/assimilation. Leaf 1 was sampled at position 3 (j) in 2013 (k) and 2014 (l) after 24 h of uptake/assimilation. The means of 3–4 replicates +/− SEM are shown. Asterisks indicate significant differences (*P* < 0.05) against the 0 N application rate, based on the Dunnett’s multiple means comparison. Dunn’s multiple means comparison was used where data was non-normal. RLU, relative light units intercepted by the luminometer in a one second interval per well. The leaf position gradient is highlighted in Additional file 3: Figure S3. (PNG 1669 kb)
Additional file 3: Figure S3. Independent trials of *GlnLux* output of maize seedling leaves using the leaf disk assay to highlight the spatial leaf position gradient. Shown is the data from 2013 (b, c, g, h) and 2014 (d, e, i, j). Leaf 3 (a-e) was sampled at position 1 and 4, and leaf 1 (f-j) was sampled at position 1 and 3 after 24 h of N uptake/assimilation with 0, 2, 5, 10, 15, or 20 mM N. The means of 3–4 replicates +/− SEM are shown. Asterisks indicate significant differences (*P* < 0.05) against the 0 N application rate, based on the Dunnett’s multiple means comparison, or Dunn’s multiple means comparison where data was non-normal. RLU, relative light units intercepted by the luminometer in a one second interval per well. The N-uptake/assimilation and temporal response gradients are highlighted in Additional file 2: Figure S2. (PNG 1157 kb)
Additional file 4: Figure S4. Comparison of leaf vein resolution between *GlnLux* *in situ* imaging and light microscopy. *GlnLux* *in situ* images (see Fig. 3) of leaves 1, 2 and 3 from plants provided with the + N treatment for 24 h of uptake/assimilation were divided into a base, middle, and tip section (1200 mm in width) equally spaced along the leaf blade (a). Leaves 1, 2, and 3 from plants (grown with only ddH_2_O in Turface® gravel until they were at the same growth stage as the main experiments) were divided in the same way for 4x brightfield microscopy (b). Diameters of the midvein tissues (c) were quantified with microscopy in NIS-Elements (version 4.51, Nikon Instruments, Tokyo, Japan). Asterisks indicate significant differences as determined with Šídák’s multiple comparison tests (*P* < 0.05) between any one base, middle or tip position and the other two positions within individual leaves. The midrib was not visible in any of the *GlnLux* images. Diameters of longitudinal (d) and transverse (e) vein tissues were quantified with *GlnLux* *in situ* image analysis in ImageJ (version 1.50i, NIH, Bethesda, USA), and with microscopy. Diameters from both quantification methods were compared at the base, middle and tip positions in all leaves with the Holm-Šídák test, and found to differ significantly (*P* < 0.05) in every *GlnLux* vs. microscopy comparison (d, e). Means of three biological replicates per leaf position composed of three subsamples +/− SEM are displayed. (PNG 136 kb)
Additional file 5: Figure S5. Visualization of luminescence produced over time by maize leaves 1, 2, and 3 (shown from left to right) when placed on *GlnLux* agar. Plants were initially germinated and grown with only ddH_2_O in Turface® gravel until they were at the same growth stage as the main experiments (eight days). Hoagland’s solution containing 20 mM N was then provided for 2 h, after which plants were moved back to N-free solution for a further 10 h. Leaves 1, 2, and 3 were harvested, freeze-thawed, and placed on *GlnLux* agar alongside disk standards of Gln (0, 3.125 × 10^−4^, 6.250 × 10^−4^, 1.250 × 10^−3^, 2.500 × 10^−3^, 5.000 × 10^−3^, 1 10^−2^ M Gln, left to right; *V* = 51 μl, r = 3 mm) (a). Plates were imaged once before incubation (b), then incubated at 37 °C for intervals of 1000 s with imaging following each interval (c-m). Plates were incubated a further 6.5 h and imaged (n). All images were captured with a 1000 s exposure and standardized to a range of 1000–6000 light intensity units. Red-yellow-green indicates diminishing *GlnLux* response, and black indicates absence of *GlnLux* output. (PNG 1339 kb)
Additional file 6: Figure S6.
*GlnLux* agar response to agar disks (radius = 3 mm) containing Gln standards (0, 3.125 × 10^−4^, 6.250 × 10^−4^, 1.250 × 10^−3^, 2.500 × 10^−3^, 5.000 × 10^−3^, 1 × 10^−2^ M Gln; C0-C6 respectively) of three different heights/volumes scaled linearly (*h* = 1.8, 3.6, 5.4 mm; *V* = 51, 102, 153 μl). Disks were placed on *GlnLux* solid agar media (a, b). Plates were then incubated at 37 °C for 2.5 h and imaged (c, d). Raw image output is shown (c) alongside the same image standardized to display a range of 1000–6000 light intensity units (d). Plates were incubated another 3.5 h and imaged (e) with standardization applied (f). White-red-yellow-green indicates diminishing *GlnLux* response, and black indicates absence of *GlnLux* output. All images were captured with a 1000 s exposure. (PNG 1672 kb)
Additional file 7: Figure S7.
*GlnLux* solid agar response to pure Gln agar disks (radius = 3 mm) with total moles of Gln per disk held constant (0, 15.94, 31.87, 63.75, 127.5, 255.0, 510.0 nmol; M0-M6 respectively) across different levels of disk height/volume (*h* = 1.8, 3.6, 5.4 mm; *V* = 51, 102, 153 μl). Disks were placed on *GlnLux* solid agar media (a, b). Plates were then incubated at 37 °C for 2.5 h and imaged (c, d). Raw image output in shown (c) alongside the same image standardized to display a range of 1000–6000 light intensity units (d). Plates were incubated another 3.5 h and imaged (e) with standardization applied (f). Red-yellow-green indicates diminishing *GlnLux* response, and black indicates absence of *GlnLux* output. All images were captured with a 1000 s exposure. (PNG 1470 kb)
Additional file 8: Figure S8. Visualization of an apparent inhibitory effect of maize seedling leaves on *GlnLux* luminescence output. Plants were initially germinated and grown with only ddH_2_O in Turface® gravel until they were at the same growth stage as the main experiments (eight days). Hoagland’s solution containing 20 mM N was then provided for 1 h. Leaf 1 was harvested, freeze-thawed, and placed on *GlnLux* agar beside sterile green paper and two disk standards (1 × 10^−2^ M Gln, volume = 51 μl) (a). Plates were imaged once prior to incubation (b), then incubated at 37 °C for intervals of 1000 s with imaging following each interval (c-m). Plates were incubated a further 6.5 h and imaged (n). All images were captured with a 1000 s exposure time and standardized to a range of 1000–6000 light intensity units. Red-yellow-green indicates diminishing *GlnLux* response, and black indicates absence of *GlnLux* output. (PNG 776 kb)
Additional file 9: Table S1. Replicate versus treatment variability of the *GlnLux in situ* imaging protocol. Three replicates of raw *GlnLux* agar plate images (Fig. 3) were analysed for each N treatment (+/-) and leaf (1-3) combination (6 plates total per leaf). A 1 x 10^-2^ M Gln agar disk was also included on each plate for standardization. The ratios of luminescence produced by each standard disk against the *GlnLux* agar background were pooled to generate SEM and an estimate of plate-to-plate variability. The luminescence output of all three replicates for each N treatment was pooled to generate SEM, and an estimate of the comparative variability due to N uptake/assimilation. Values represent the SEM of 6 plates each. Significant difference at *P*<0.05 between the variance of the standardization ratio and leaf luminescence is indicated with an asterisk, as determined with *F* tests. Quantification of luminescence was performed using WinView software (version 2.5.16.5, Princeton Instruments, Trenton, USA). (DOCX 43 kb)

## Abbreviations

Gln: Glutamine
N: Nitrogen
NUE: Nitrogen use efficiency
