# Supplementary figures and images for: Biosensor-based spatial and developmental mapping of maize leaf glutamine at vein-level resolution in response to different nitrogen rates and uptake/assimilation durations
Source: BMC Plant Biol. 2016 Oct 21;16:230. doi: 10.1186/s12870-016-0918-x (PMC5075184; doi:10.1186/s12870-016-0918-x)

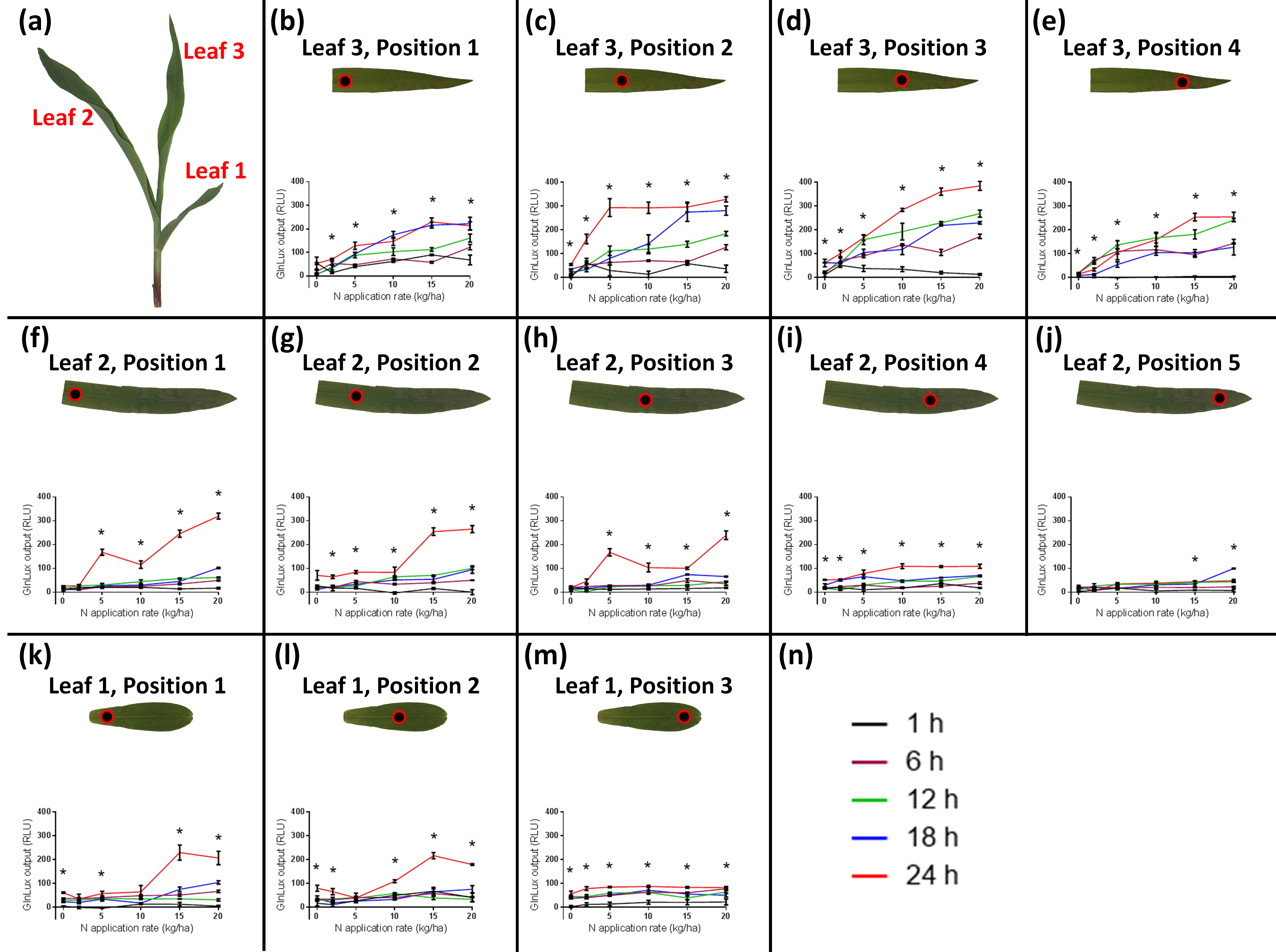

Supplement: Additional file 1: Figure S1. — Gradients of GlnLux output of leaves of maize seedlings using the GlnLux leaf disk assay. Leaves 1, 2 and 3 were sampled (a). Leaf 3 was assayed at positions 1 (b), 2 (c), 3 (d), and 4 (e), extending from the leaf base to leaf tip. Leaf 2 was assayed at positions 1 (f), 2 (g), 3 (h), 4 (i), and 5 (j). Leaf 1 was assayed at positions 1 (k), 2 (l), and 3 (m). Plants had not been provided with N from germination for a period of 12 days, at which time modified Hoagland’s solution containing 0, 2, 5, 10, 15 or 20 mM N was applied. Plants were allowed different durations (1, 6, 12, 18 or 24 h) of N uptake/assimilation (n), after which tissue disks were harvested. Means of 3–4 replicates +/− SEM are shown. RLU, relative light units intercepted by the luminometer in a one second interval per well. Asterisks indicate significant differences between the 1 h (black lines) and 24 h (red lines) treatments, at different N application rates, based on the Holm-Šídák test at P < 0.05. The data is displayed to highlight the N rate response gradient. The N uptake/assimilation gradient is highlighted in Fig. 2. The two datasets are the same. Shown is Trial 1. For Trial 2, see Additional file 2: Figure S2 and Additional file 3: Figure S3. (PNG 4607 kb) [file 12870_2016_918_MOESM1_ESM.png]

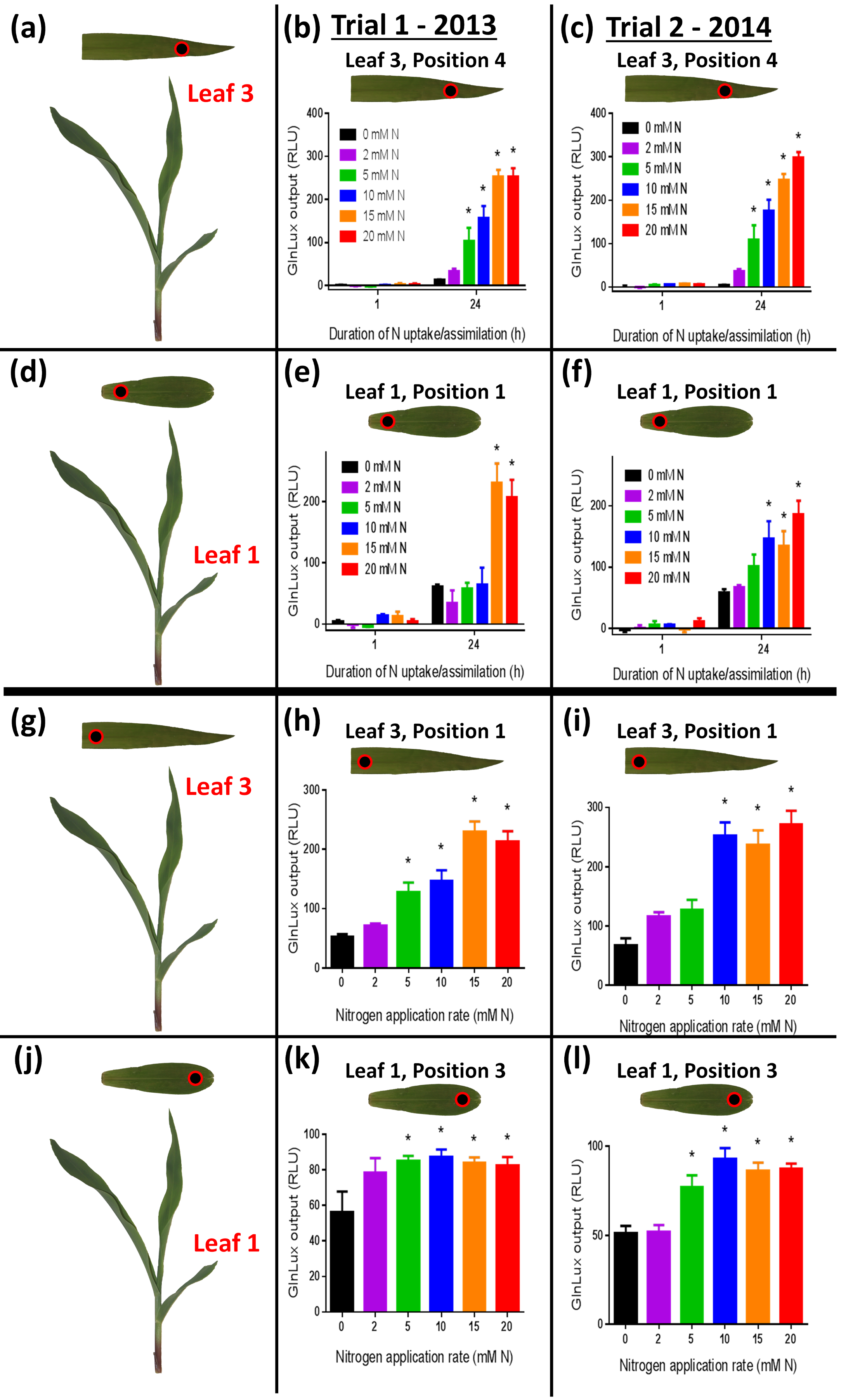

Supplement: Additional file 2: Figure S2. — Independent trials of GlnLux output of maize seedling leaves using the leaf disk assay. Shown is the data from Trial 1 (b, e, h, k) and Trial 2 (c, f, i, l). Two time points of N-uptake and assimilation (1, 24 h) are shown in panels (a-f) to highlight the temporal response gradient, while in panels (g-l) a single time point (24 h) is shown to highlight the N rate response gradient. Leaf 3 was sampled at position 4 (a) in 2013 (b) and 2014 (c), after 1 and 24 h of uptake/assimilation of 0, 2, 5, 10, 15, or 20 mM N. Leaf 1 was sampled at position 1 (d) in 2013 (e) and 2014 (f) after 1 and 24 h of uptake/assimilation. Leaf 3 was sampled at position 1 (g) in 2013 (h) and 2014 (i) after 24 h of uptake/assimilation. Leaf 1 was sampled at position 3 (j) in 2013 (k) and 2014 (l) after 24 h of uptake/assimilation. The means of 3–4 replicates +/− SEM are shown. Asterisks indicate significant differences (P < 0.05) against the 0 N application rate, based on the Dunnett’s multiple means comparison. Dunn’s multiple means comparison was used where data was non-normal. RLU, relative light units intercepted by the luminometer in a one second interval per well. The leaf position gradient is highlighted in Additional file 3: Figure S3. (PNG 1669 kb) [file 12870_2016_918_MOESM2_ESM.png]

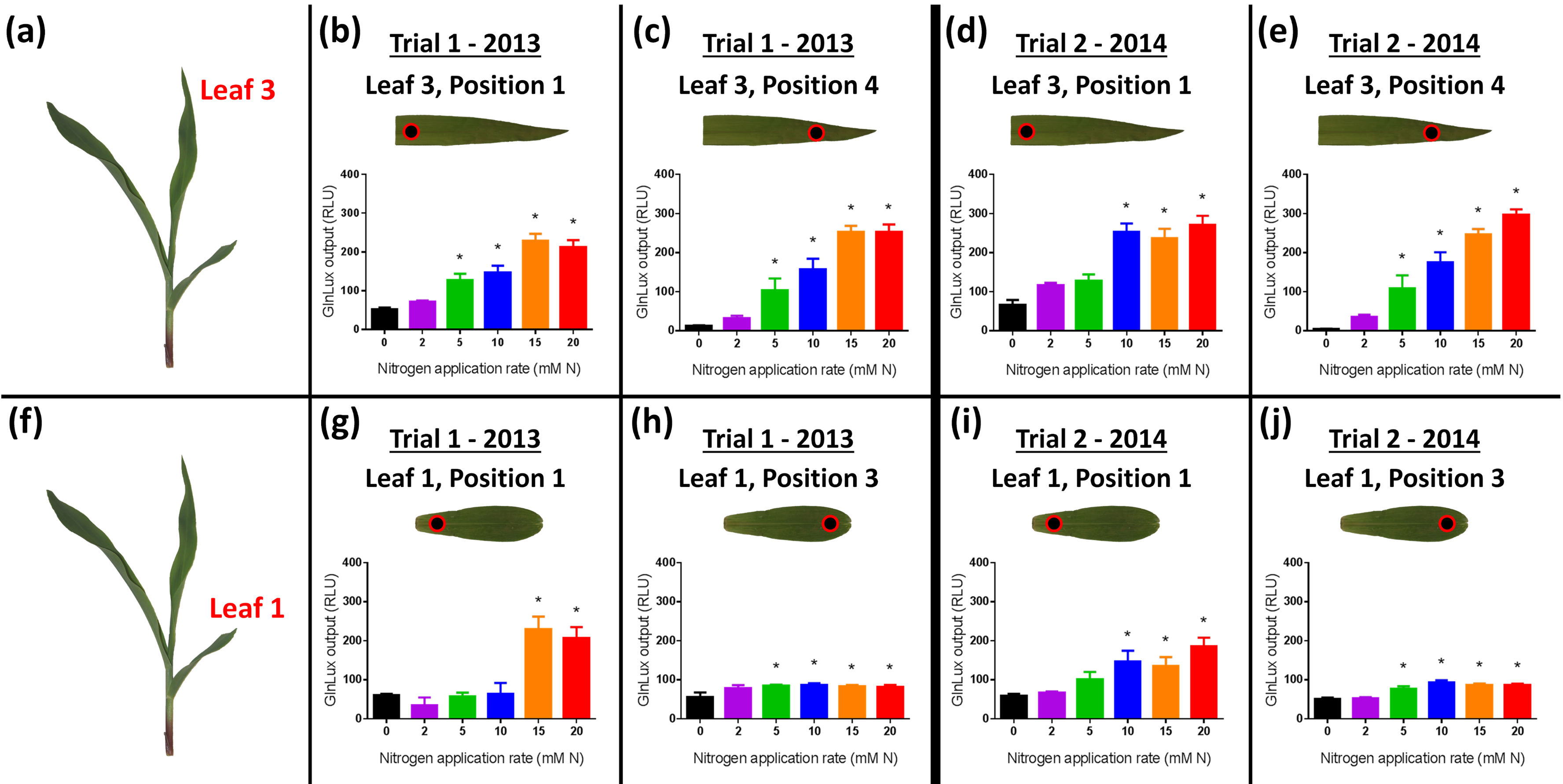

Supplement: Additional file 3: Figure S3. — Independent trials of GlnLux output of maize seedling leaves using the leaf disk assay to highlight the spatial leaf position gradient. Shown is the data from 2013 (b, c, g, h) and 2014 (d, e, i, j). Leaf 3 (a-e) was sampled at position 1 and 4, and leaf 1 (f-j) was sampled at position 1 and 3 after 24 h of N uptake/assimilation with 0, 2, 5, 10, 15, or 20 mM N. The means of 3–4 replicates +/− SEM are shown. Asterisks indicate significant differences (P < 0.05) against the 0 N application rate, based on the Dunnett’s multiple means comparison, or Dunn’s multiple means comparison where data was non-normal. RLU, relative light units intercepted by the luminometer in a one second interval per well. The N-uptake/assimilation and temporal response gradients are highlighted in Additional file 2: Figure S2. (PNG 1157 kb) [file 12870_2016_918_MOESM3_ESM.png]

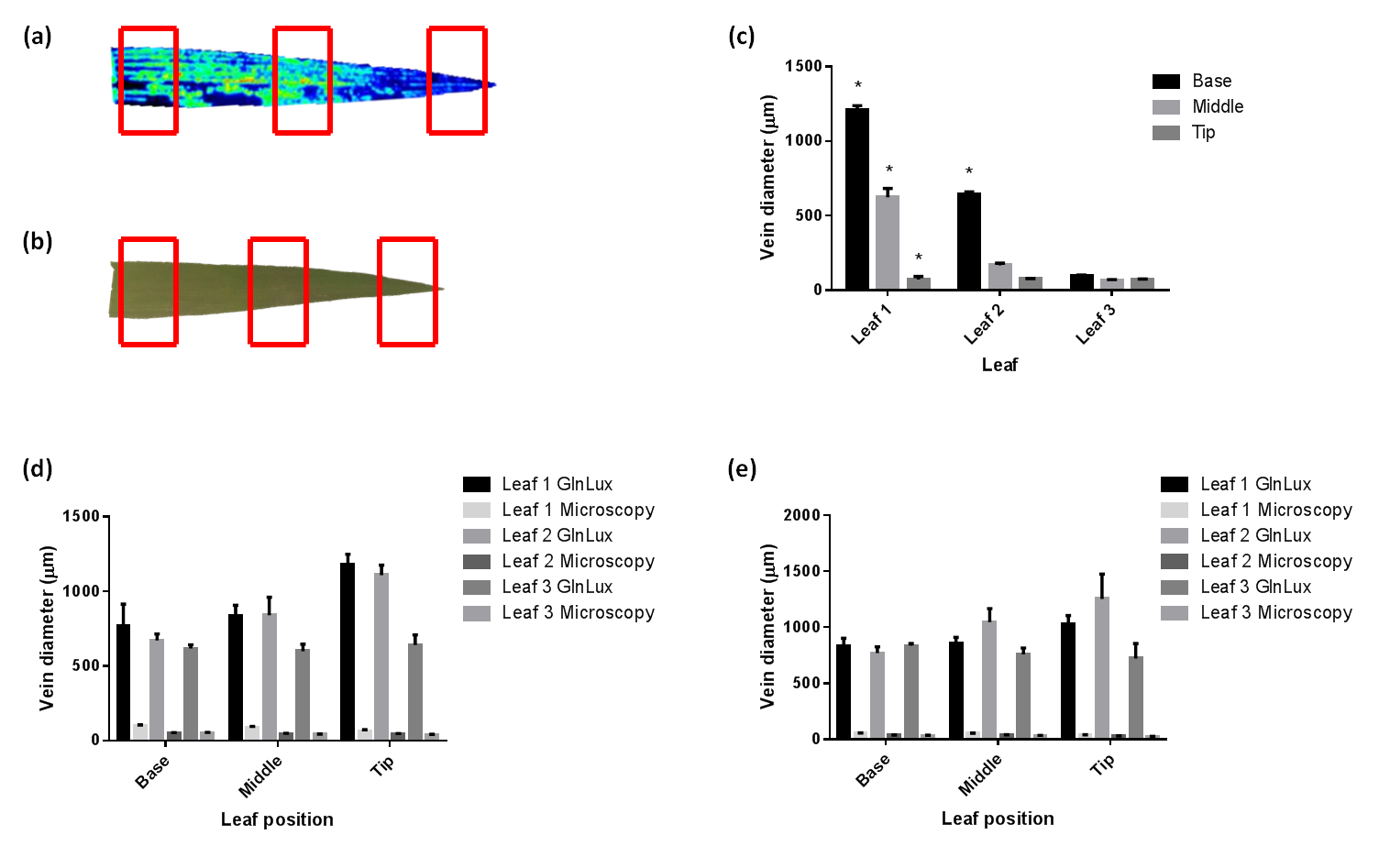

Supplement: Additional file 4: Figure S4. — Comparison of leaf vein resolution between GlnLux in situ imaging and light microscopy. GlnLux in situ images (see Fig. 3) of leaves 1, 2 and 3 from plants provided with the + N treatment for 24 h of uptake/assimilation were divided into a base, middle, and tip section (1200 mm in width) equally spaced along the leaf blade (a). Leaves 1, 2, and 3 from plants (grown with only ddH2O in Turface® gravel until they were at the same growth stage as the main experiments) were divided in the same way for 4x brightfield microscopy (b). Diameters of the midvein tissues (c) were quantified with microscopy in NIS-Elements (version 4.51, Nikon Instruments, Tokyo, Japan). Asterisks indicate significant differences as determined with Šídák’s multiple comparison tests (P < 0.05) between any one base, middle or tip position and the other two positions within individual leaves. The midrib was not visible in any of the GlnLux images. Diameters of longitudinal (d) and transverse (e) vein tissues were quantified with GlnLux in situ image analysis in ImageJ (version 1.50i, NIH, Bethesda, USA), and with microscopy. Diameters from both quantification methods were compared at the base, middle and tip positions in all leaves with the Holm-Šídák test, and found to differ significantly (P < 0.05) in every GlnLux vs. microscopy comparison (d, e). Means of three biological replicates per leaf position composed of three subsamples +/− SEM are displayed. (PNG 136 kb) [file 12870_2016_918_MOESM4_ESM.png]

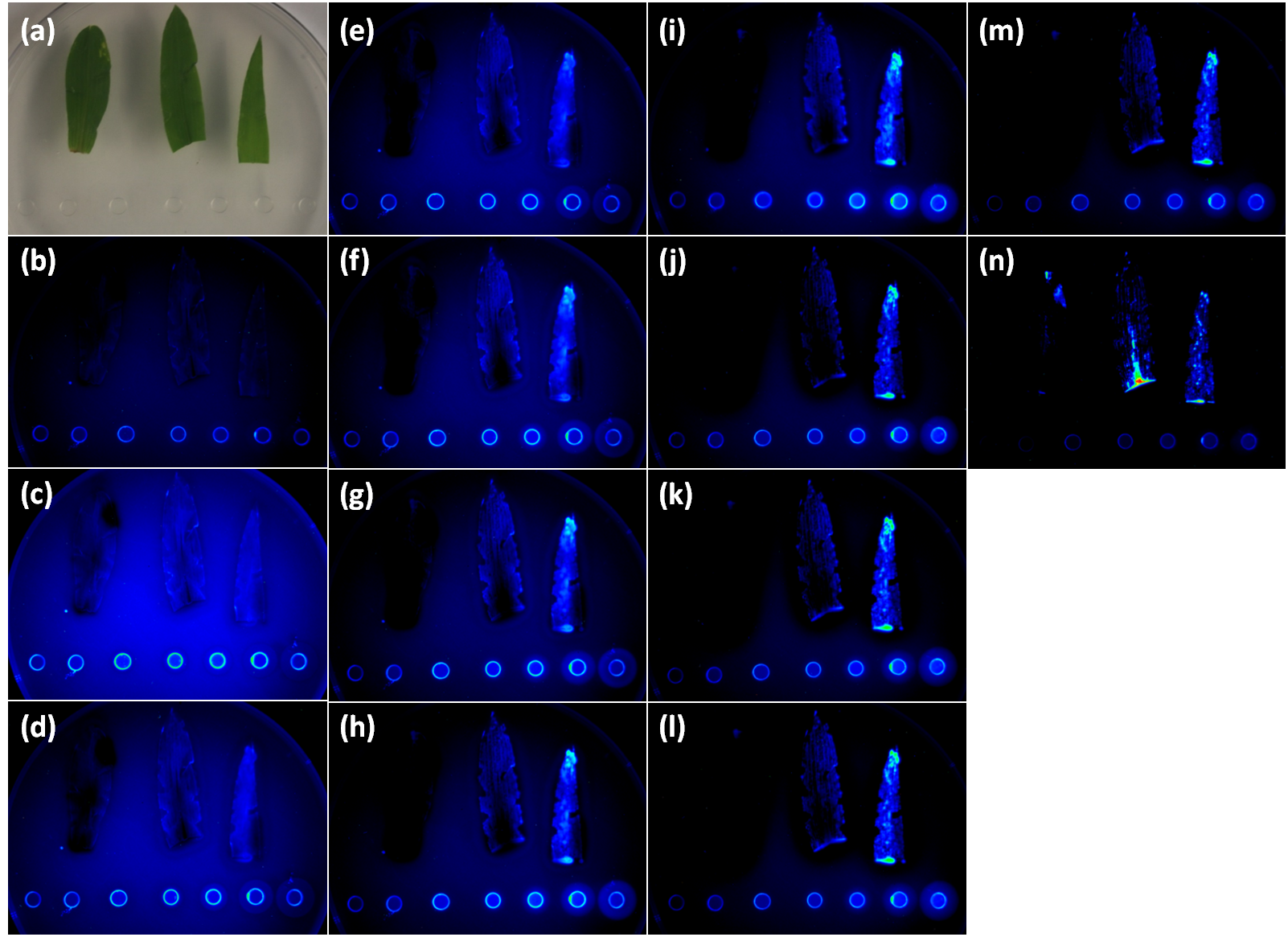

Supplement: Additional file 5: Figure S5. — Visualization of luminescence produced over time by maize leaves 1, 2, and 3 (shown from left to right) when placed on GlnLux agar. Plants were initially germinated and grown with only ddH2O in Turface® gravel until they were at the same growth stage as the main experiments (eight days). Hoagland’s solution containing 20 mM N was then provided for 2 h, after which plants were moved back to N-free solution for a further 10 h. Leaves 1, 2, and 3 were harvested, freeze-thawed, and placed on GlnLux agar alongside disk standards of Gln (0, 3.125 × 10^−4^, 6.250 × 10^−4^, 1.250 × 10^−3^, 2.500 × 10^−3^, 5.000 × 10^−3^, 1 10−2 M Gln, left to right; V = 51 μl, r = 3 mm) (a). Plates were imaged once before incubation (b), then incubated at 37 °C for intervals of 1000 s with imaging following each interval (c-m). Plates were incubated a further 6.5 h and imaged (n). All images were captured with a 1000 s exposure and standardized to a range of 1000–6000 light intensity units. Red-yellow-green indicates diminishing GlnLux response, and black indicates absence of GlnLux output. (PNG 1339 kb) [file 12870_2016_918_MOESM5_ESM.png]

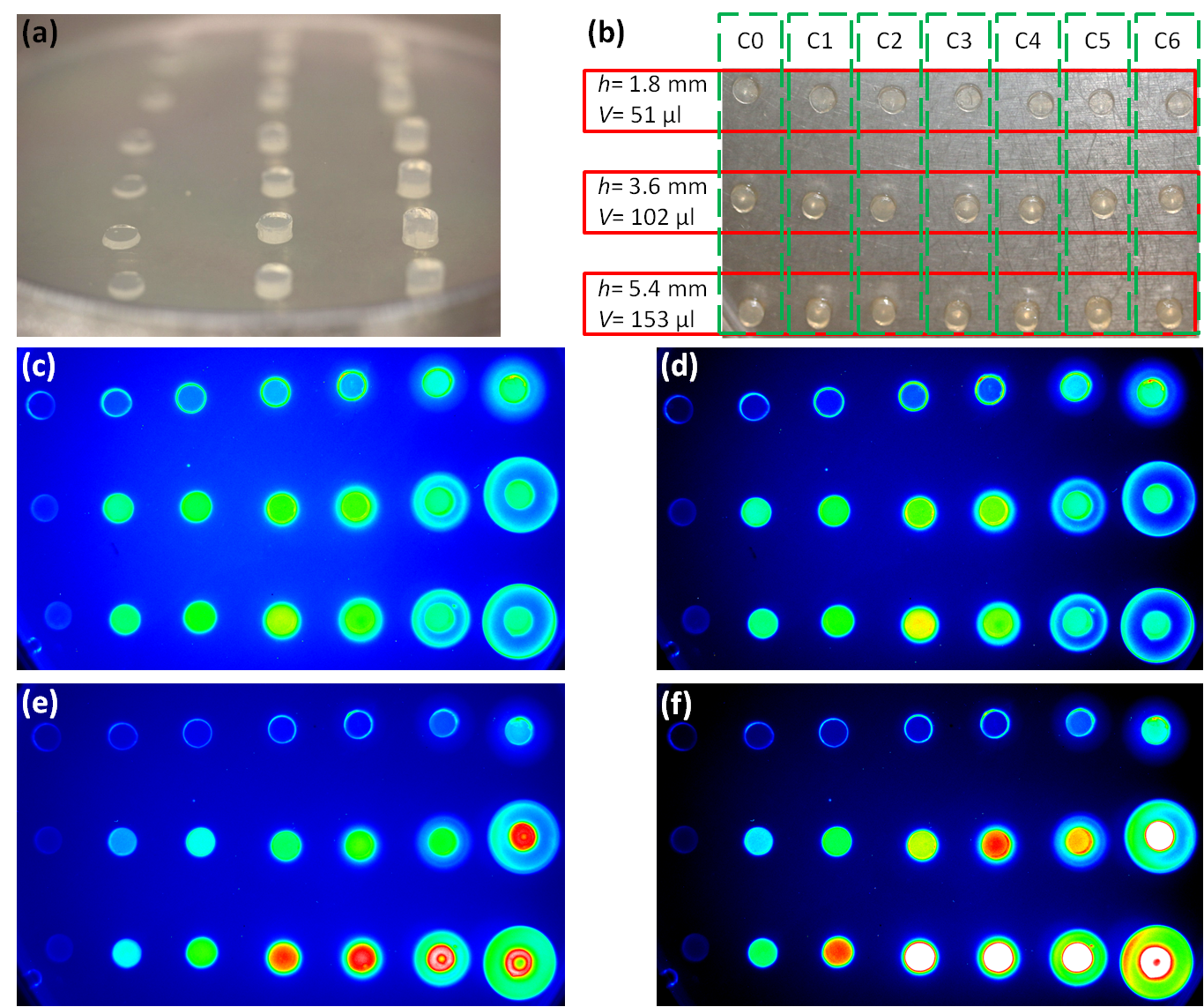

Supplement: Additional file 6: Figure S6. — GlnLux agar response to agar disks (radius = 3 mm) containing Gln standards (0, 3.125 × 10^−4^, 6.250 × 10^−4^, 1.250 × 10^−3^, 2.500 × 10^−3^, 5.000 × 10^−3^, 1 × 10^−2^ M Gln; C0-C6 respectively) of three different heights/volumes scaled linearly (h = 1.8, 3.6, 5.4 mm; V = 51, 102, 153 μl). Disks were placed on GlnLux solid agar media (a, b). Plates were then incubated at 37 °C for 2.5 h and imaged (c, d). Raw image output is shown (c) alongside the same image standardized to display a range of 1000–6000 light intensity units (d). Plates were incubated another 3.5 h and imaged (e) with standardization applied (f). White-red-yellow-green indicates diminishing GlnLux response, and black indicates absence of GlnLux output. All images were captured with a 1000 s exposure. (PNG 1672 kb) [file 12870_2016_918_MOESM6_ESM.png]

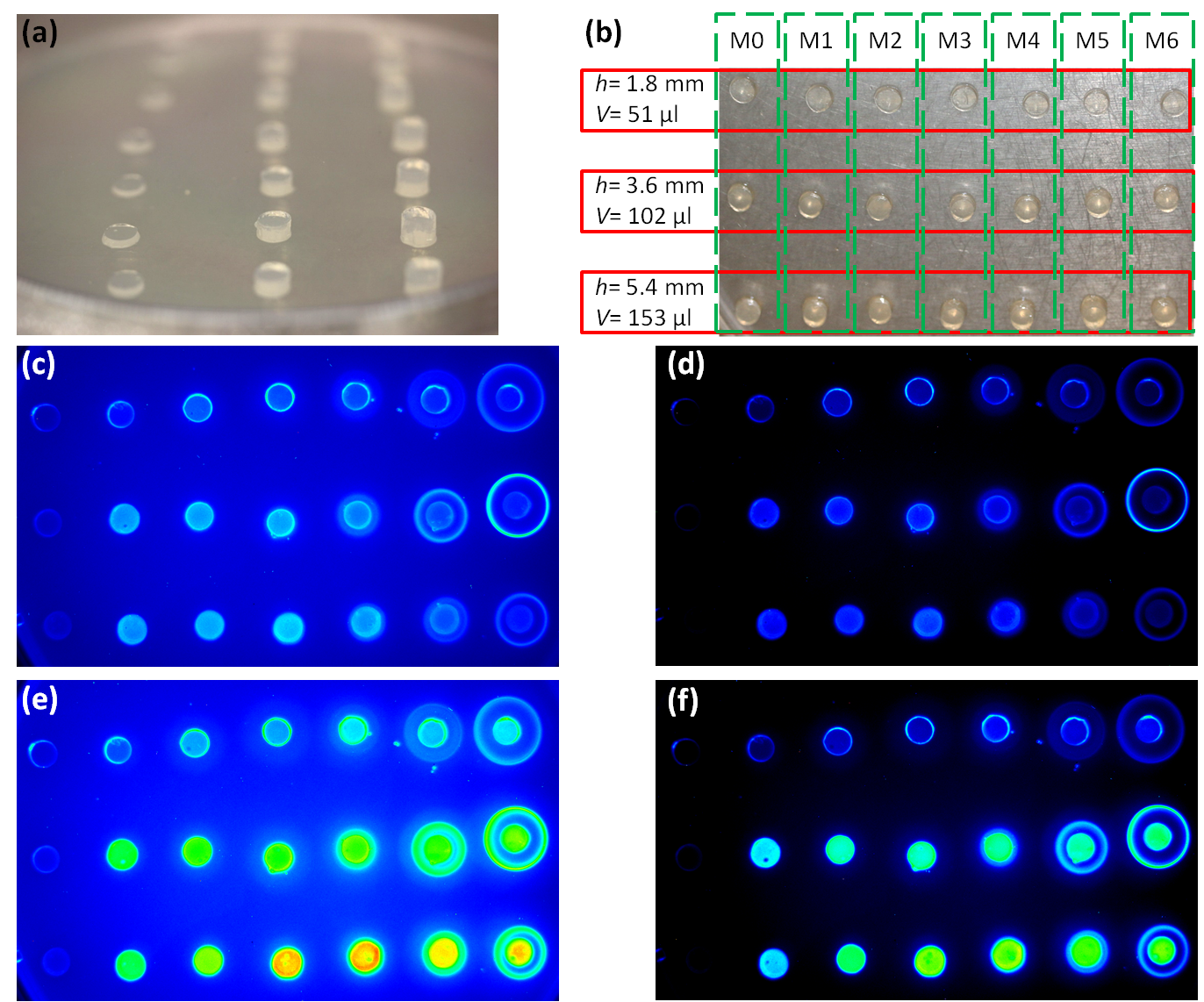

Supplement: Additional file 7: Figure S7. — GlnLux solid agar response to pure Gln agar disks (radius = 3 mm) with total moles of Gln per disk held constant (0, 15.94, 31.87, 63.75, 127.5, 255.0, 510.0 nmol; M0-M6 respectively) across different levels of disk height/volume (h = 1.8, 3.6, 5.4 mm; V = 51, 102, 153 μl). Disks were placed on GlnLux solid agar media (a, b). Plates were then incubated at 37 °C for 2.5 h and imaged (c, d). Raw image output in shown (c) alongside the same image standardized to display a range of 1000–6000 light intensity units (d). Plates were incubated another 3.5 h and imaged (e) with standardization applied (f). Red-yellow-green indicates diminishing GlnLux response, and black indicates absence of GlnLux output. All images were captured with a 1000 s exposure. (PNG 1470 kb) [file 12870_2016_918_MOESM7_ESM.png]

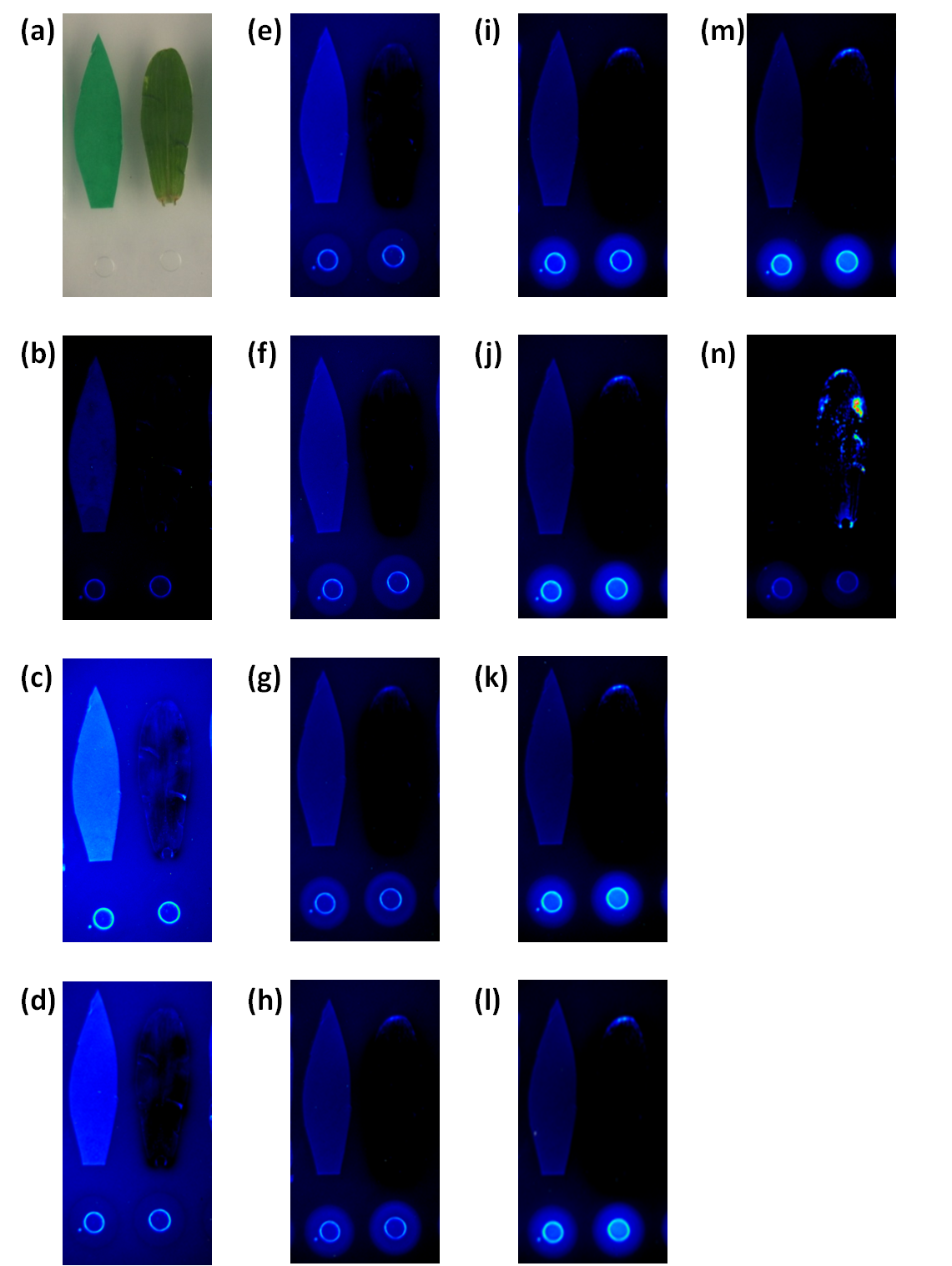

Supplement: Additional file 8: Figure S8. — Visualization of an apparent inhibitory effect of maize seedling leaves on GlnLux luminescence output. Plants were initially germinated and grown with only ddH2O in Turface® gravel until they were at the same growth stage as the main experiments (eight days). Hoagland’s solution containing 20 mM N was then provided for 1 h. Leaf 1 was harvested, freeze-thawed, and placed on GlnLux agar beside sterile green paper and two disk standards (1 × 10^−2^ M Gln, volume = 51 μl) (a). Plates were imaged once prior to incubation (b), then incubated at 37 °C for intervals of 1000 s with imaging following each interval (c-m). Plates were incubated a further 6.5 h and imaged (n). All images were captured with a 1000 s exposure time and standardized to a range of 1000–6000 light intensity units. Red-yellow-green indicates diminishing GlnLux response, and black indicates absence of GlnLux output. (PNG 776 kb) [file 12870_2016_918_MOESM8_ESM.png]
