# Supplementary material for: Biosensor-based spatial and developmental mapping of maize leaf glutamine at vein-level resolution in response to different nitrogen rates and uptake/assimilation durations
Source: BMC Plant Biol. 2016 Oct 21;16:230. doi: 10.1186/s12870-016-0918-x (PMC5075184; doi:10.1186/s12870-016-0918-x)
Supplement: Additional file 9: Table S1. — Replicate versus treatment variability of the GlnLux in situ imaging protocol. Three replicates of raw GlnLux agar plate images (Fig. 3) were analysed for each N treatment (+/-) and leaf (1-3) combination (6 plates total per leaf). A 1 x 10^-2^ M Gln agar disk was also included on each plate for standardization. The ratios of luminescence produced by each standard disk against the GlnLux agar background were pooled to generate SEM and an estimate of plate-to-plate variability. The luminescence output of all three replicates for each N treatment was pooled to generate SEM, and an estimate of the comparative variability due to N uptake/assimilation. Values represent the SEM of 6 plates each. Significant difference at P<0.05 between the variance of the standardization ratio and leaf luminescence is indicated with an asterisk, as determined with F tests. Quantification of luminescence was performed using WinView software (version 2.5.16.5, Princeton Instruments, Trenton, USA). (DOCX 43 kb) [file 12870_2016_918_MOESM9_ESM.docx]

**Additional File 9: Table S1.** Replicate versus treatment variability of the *GlnLux* *in situ* imaging protocol. Three replicates of raw *GlnLux* agar plate images (Fig. 3) were analysed for each N treatment (+/ -) and leaf (1-3) combination (6 plates total per leaf). A 1 x 10^-2^ M Gln agar disk was also included on each plate for standardization. The ratios of luminescence produced by each standard disk against the *GlnLux* agar background were pooled to generate SEM and an estimate of plate-to-plate variability. The luminescence output of all three replicates for each N treatment was pooled to generate SEM, and an estimate of the comparative variability due to N uptake/assimilation. Values represent the SEM of 6 plates each. Significant difference at P<0.05 between the variance of the standardization ratio and leaf luminescence is indicated with an asterisk, as determined with F tests. Quantification of luminescence was performed using WinView software (version 2.5.16.5, Princeton Instruments, Trenton, USA).

|  |  | Leaf luminescence following N uptake/assimilation | | |
| --- | --- | --- | --- | --- |
| Leaf | Standardization ratio | 1 h | 12 h | 24 h |
| 1 | 0.1709 | 45.54* | 46.07* | 46.87* |
| 2 | 0.1528 | 30.85* | 49.58* | 117.6* |
| 3 | 0.0852 | 27.42* | 74.24* | 135.1* |
